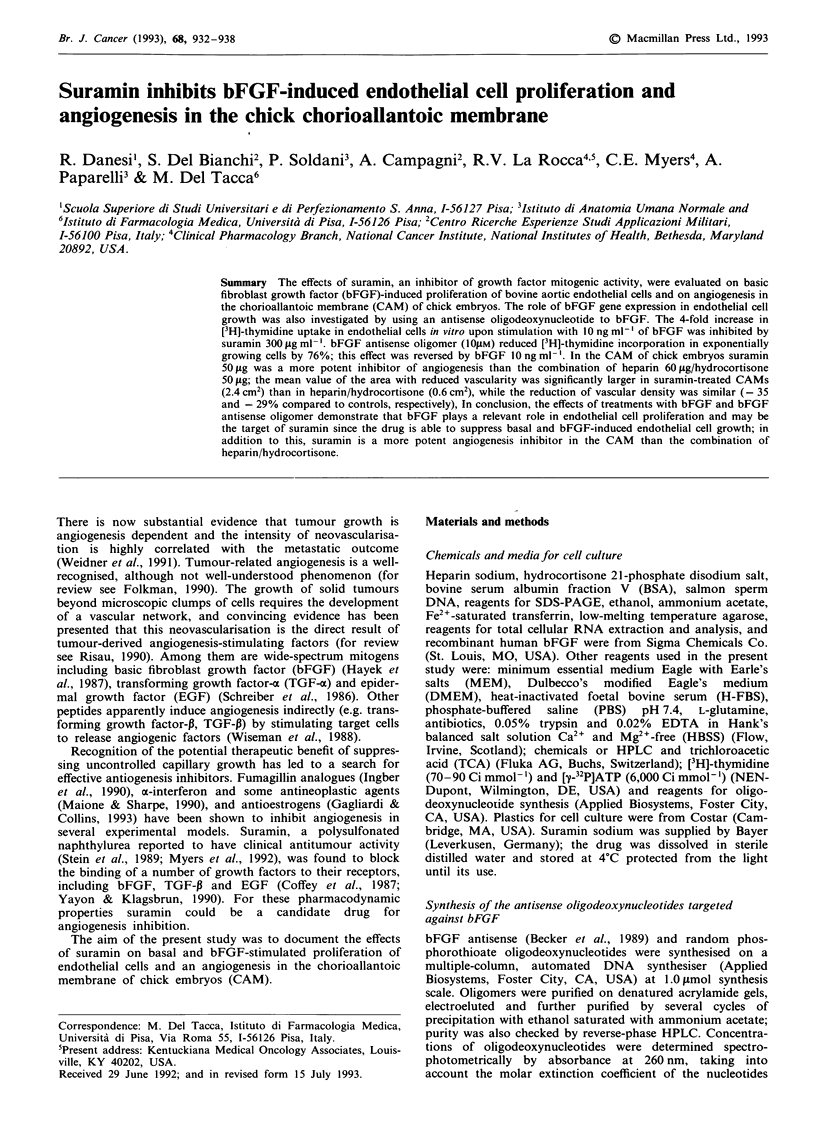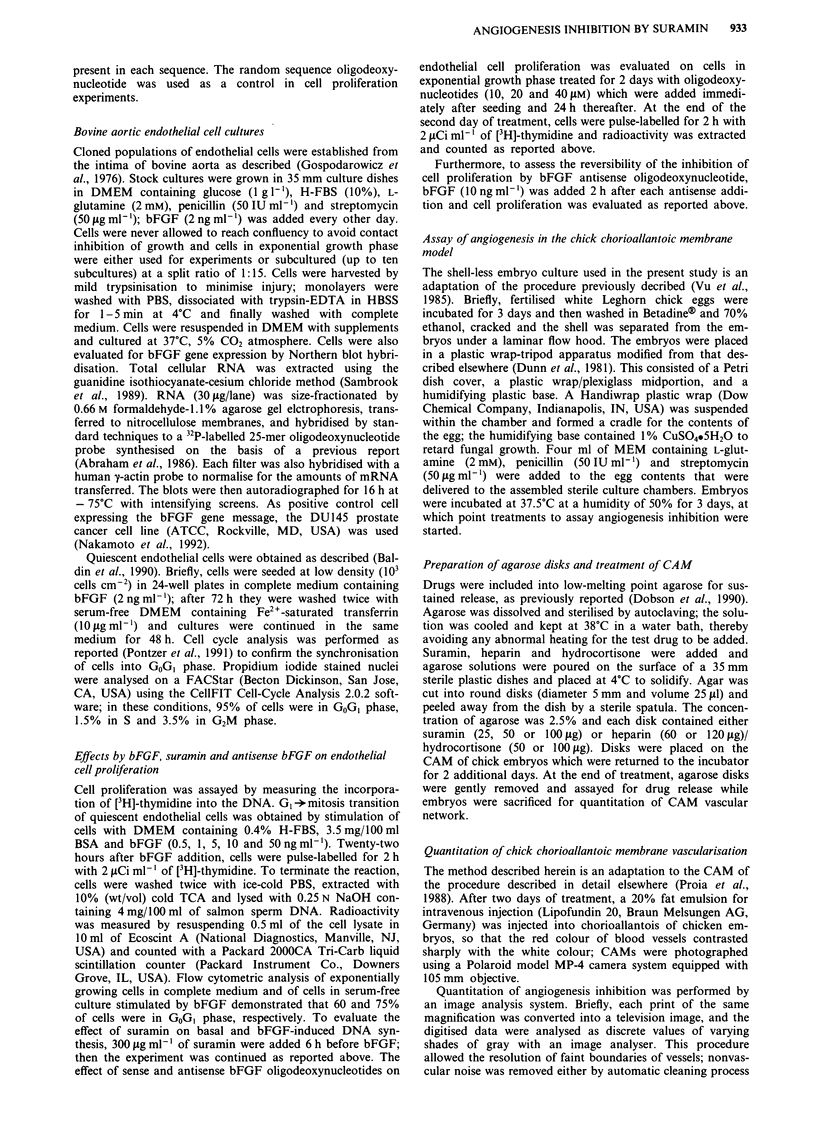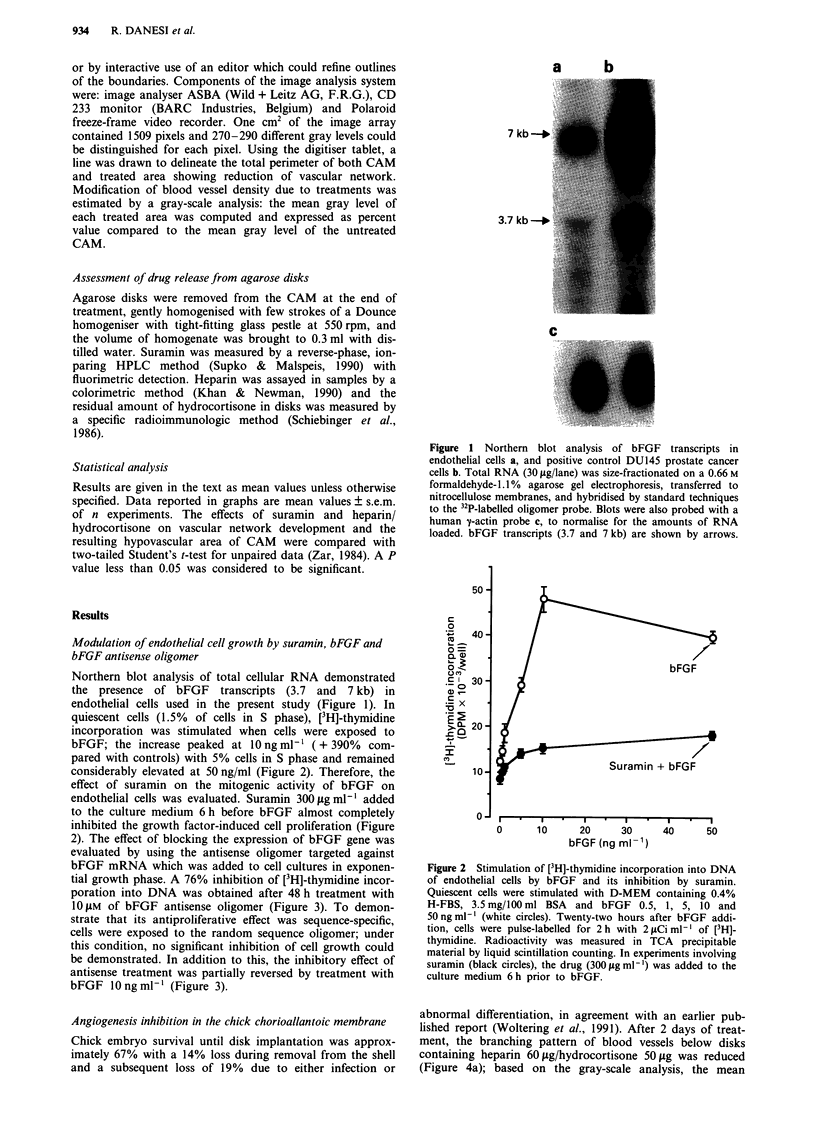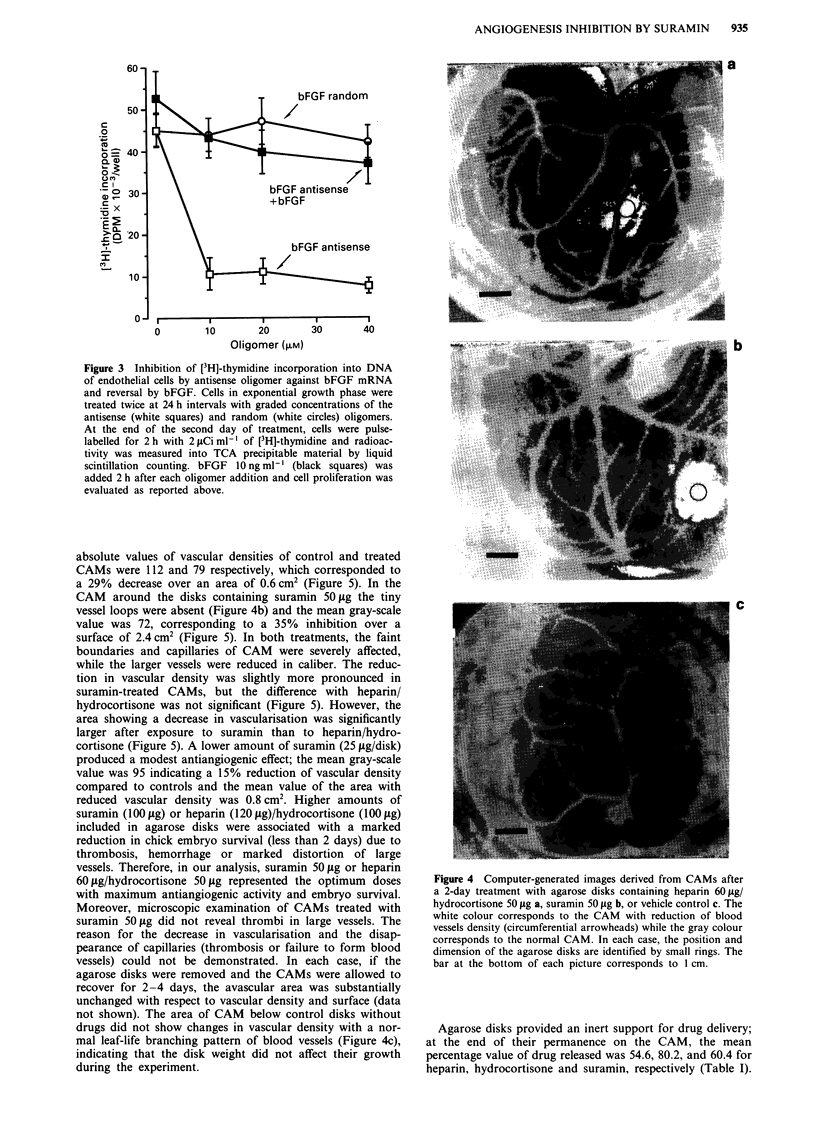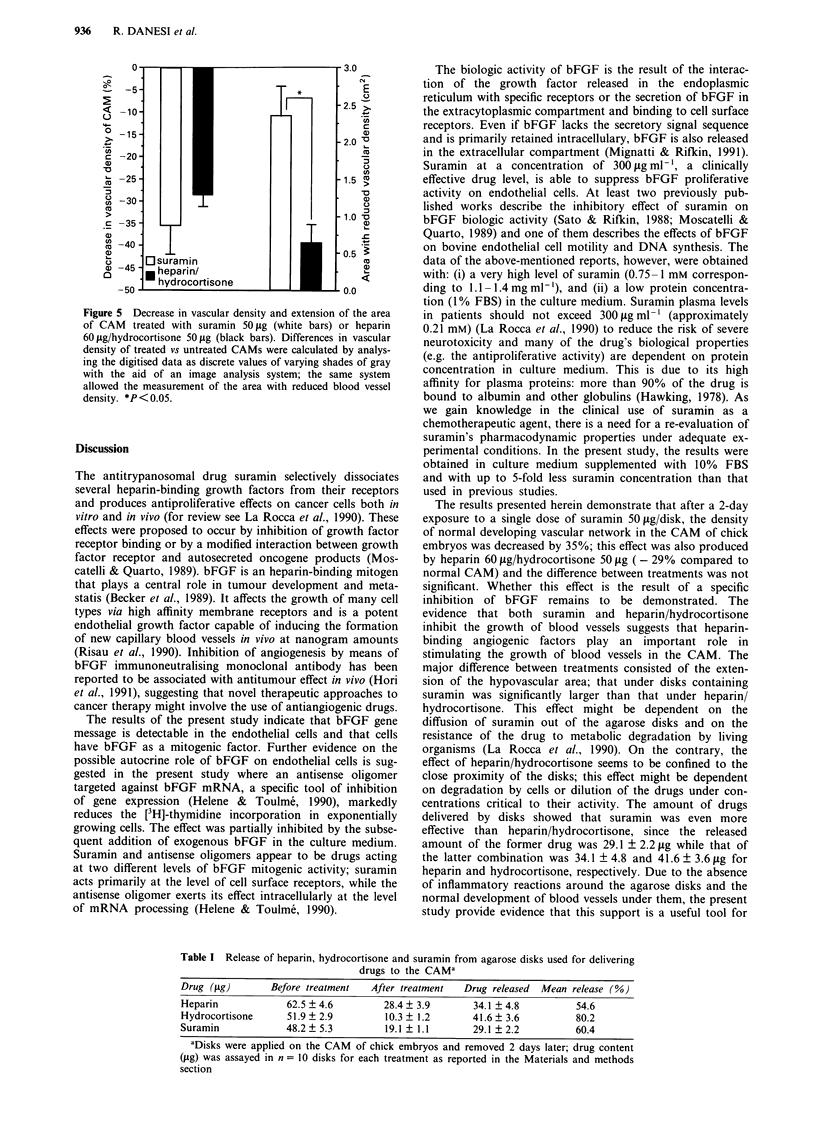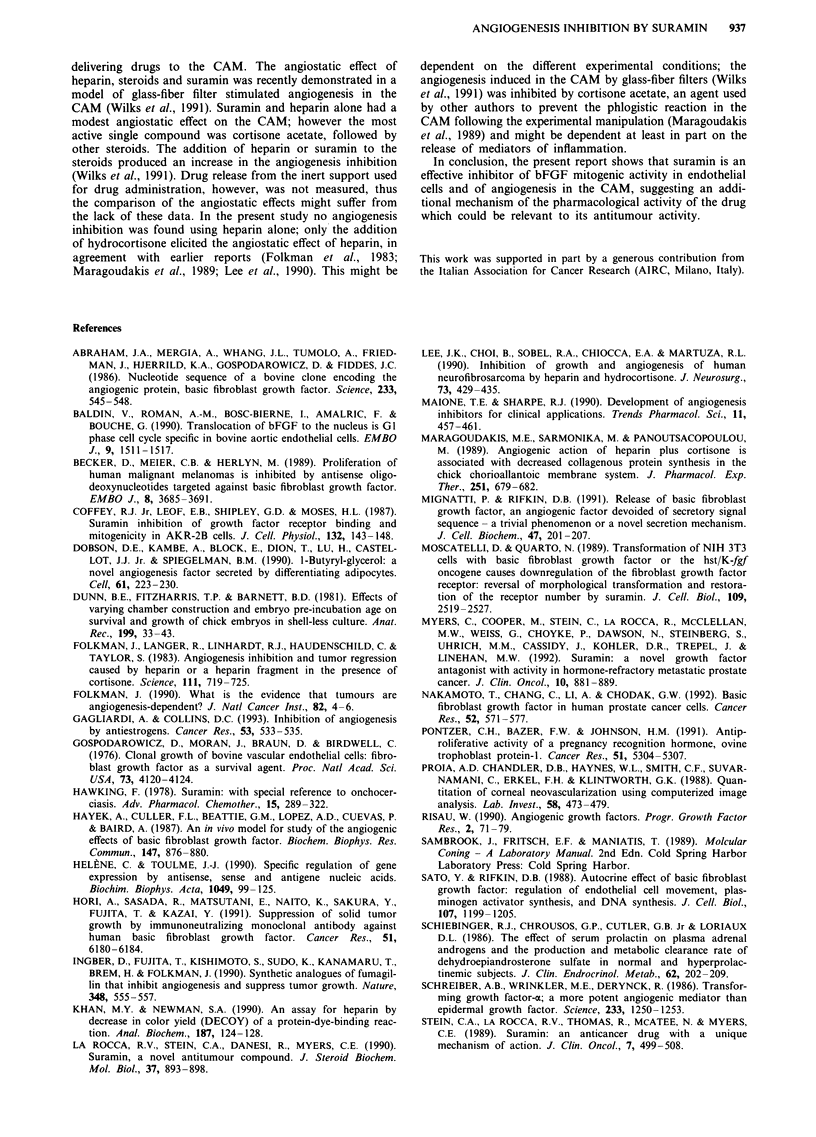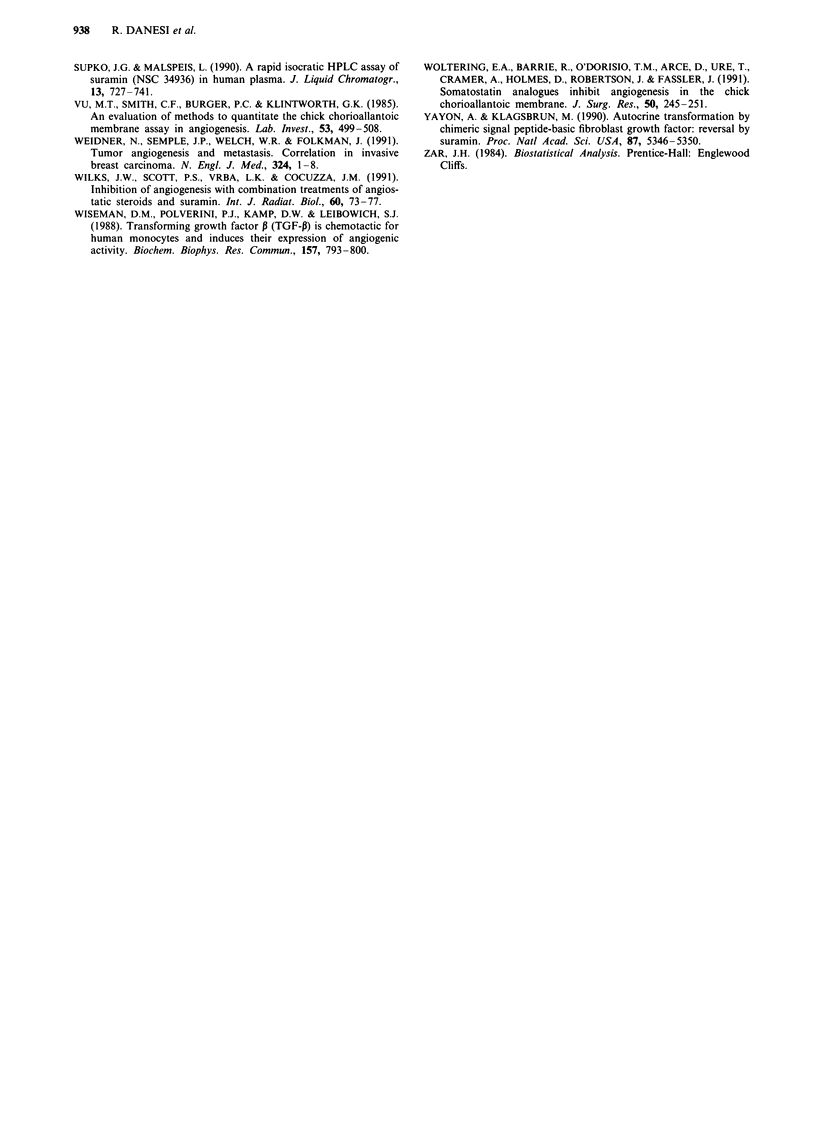# Suramin inhibits bFGF-induced endothelial cell proliferation and angiogenesis in the chick chorioallantoic membrane.

**DOI:** 10.1038/bjc.1993.457

**Published:** 1993-11

**Authors:** R. Danesi, S. Del Bianchi, P. Soldani, A. Campagni, R. V. La Rocca, C. E. Myers, A. Paparelli, M. Del Tacca

**Affiliations:** Scuola Superiore di Studi Universitari e di Perfezionamento S. Anna, Pisa, Italy.

## Abstract

**Images:**


Br. J. Cancer (1993), 68, 932–938 © Macmillan Press Ltd., 1993

### Suramin inhibits bFGF-induced endothelial cell proliferation and angiogenesis in the chick chorioallantoic membrane

R. Danesi[1], S. Del Bianchi[2], P. Soldani[3], A. Campagni[2], R.V. La Rocca[4,5], C.E. Myers[4], A. Paparelli[3] & M. Del Tacca[6]

[1]*Scuola Superiore di Studi Universitari e di Perfezionamento S. Anna, I-56127 Pisa;* [3]*Istituto di Anatomia Umana Normale and* [6]*Istituto di Farmacologia Medica, Università di Pisa, I-56126 Pisa;* [2]*Centro Ricerche Esperienze Studi Applicazioni Militari, I-56100 Pisa, Italy;* [4]*Clinical Pharmacology Branch, National Cancer Institute, National Institutes of Health, Bethesda, Maryland 20892, USA.*

**Summary** The effects of suramin, an inhibitor of growth factor mitogenic activity, were evaluated on basic fibroblast growth factor (bFGF)-induced proliferation of bovine aortic endothelial cells and on angiogenesis in the chorioallantoic membrane (CAM) of chick embryos. The role of bFGF gene expression in endothelial cell growth was also investigated by using an antisense oligodeoxynucleotide to bFGF. The 4-fold increase in [$^3$H]-thymidine uptake in endothelial cells *in vitro* upon stimulation with 10 ng ml$^{-1}$ of bFGF was inhibited by suramin 300 µg ml$^{-1}$. bFGF antisense oligomer (10µM) reduced [$^3$H]-thymidine incorporation in exponentially growing cells by 76%; this effect was reversed by bFGF 10 ng ml$^{-1}$. In the CAM of chick embryos suramin 50 µg was a more potent inhibitor of angiogenesis than the combination of heparin 60 µg/hydrocortisone 50 µg; the mean value of the area with reduced vascularity was significantly larger in suramin-treated CAMs (2.4 cm$^2$) than in heparin/hydrocortisone (0.6 cm$^2$), while the reduction of vascular density was similar (− 35 and − 29% compared to controls, respectively), In conclusion, the effects of treatments with bFGF and bFGF antisense oligomer demonstrate that bFGF plays a relevant role in endothelial cell proliferation and may be the target of suramin since the drug is able to suppress basal and bFGF-induced endothelial cell growth; in addition to this, suramin is a more potent angiogenesis inhibitor in the CAM than the combination of heparin/hydrocortisone.

There is now substantial evidence that tumour growth is angiogenesis dependent and the intensity of neovascularisation is highly correlated with the metastatic outcome (Weidner *et al.*, 1991). Tumour-related angiogenesis is a well-recognised, although not well-understood phenomenon (for review see Folkman, 1990). The growth of solid tumours beyond microscopic clumps of cells requires the development of a vascular network, and convincing evidence has been presented that this neovascularisation is the direct result of tumour-derived angiogenesis-stimulating factors (for review see Risau, 1990). Among them are wide-spectrum mitogens including basic fibroblast growth factor (bFGF) (Hayek *et al.*, 1987), transforming growth factor-α (TGF-α) and epidermal growth factor (EGF) (Schreiber *et al.*, 1986). Other peptides apparently induce angiogenesis indirectly (e.g. transforming growth factor-β, TGF-β) by stimulating target cells to release angiogenic factors (Wiseman *et al.*, 1988).

Recognition of the potential therapeutic benefit of suppressing uncontrolled capillary growth has led to a search for effective antiogenesis inhibitors. Fumagillin analogues (Ingber *et al.*, 1990), α-interferon and some antineoplastic agents (Maione & Sharpe, 1990), and antioestrogens (Gagliardi & Collins, 1993) have been shown to inhibit angiogenesis in several experimental models. Suramin, a polysulfonated naphthylurea reported to have clinical antitumour activity (Stein *et al.*, 1989; Myers *et al.*, 1992), was found to block the binding of a number of growth factors to their receptors, including bFGF, TGF-β and EGF (Coffey *et al.*, 1987; Yayon & Klagsbrun, 1990). For these pharmacodynamic properties suramin could be a candidate drug for angiogenesis inhibition.

The aim of the present study was to document the effects of suramin on basal and bFGF-stimulated proliferation of endothelial cells and an angiogenesis in the chorioallantoic membrane of chick embryos (CAM).

Correspondence: M. Del Tacca, Istituto di Farmacologia Medica, Università di Pisa, Via Roma 55, I-56126 Pisa, Italy.
[5]Present address: Kentuckiana Medical Oncology Associates, Louisville, KY 40202, USA.
Received 29 June 1992; and in revised form 15 July 1993.

### Materials and methods

### *Chemicals and media for cell culture*

Heparin sodium, hydrocortisone 21-phosphate disodium salt, bovine serum albumin fraction V (BSA), salmon sperm DNA, reagents for SDS-PAGE, ethanol, ammonium acetate, $Fe^{2+}$-saturated transferrin, low-melting temperature agarose, reagents for total cellular RNA extraction and analysis, and recombinant human bFGF were from Sigma Chemicals Co. (St. Louis, MO, USA). Other reagents used in the present study were: minimum essential medium Eagle with Earle's salts (MEM), Dulbecco's modified Eagle's medium (DMEM), heat-inactivated foetal bovine serum (H-FBS), phosphate-buffered saline (PBS) pH 7.4, L-glutamine, antibiotics, 0.05% trypsin and 0.02% EDTA in Hank's balanced salt solution $Ca^{2+}$ and $Mg^{2+}$-free (HBSS) (Flow, Irvine, Scotland); chemicals or HPLC and trichloroacetic acid (TCA) (Fluka AG, Buchs, Switzerland); [$^3$H]-thymidine (70–90 Ci mmol$^{-1}$) and [γ-$^{32}$P]ATP (6,000 Ci mmol$^{-1}$) (NEN-Dupont, Wilmington, DE, USA) and reagents for oligodeoxynucleotide synthesis (Applied Biosystems, Foster City, CA, USA). Plastics for cell culture were from Costar (Cambridge, MA, USA). Suramin sodium was supplied by Bayer (Leverkusen, Germany); the drug was dissolved in sterile distilled water and stored at 4°C protected from the light until its use.

### *Synthesis of the antisense oligodeoxynucleotides targeted against bFGF*

bFGF antisense (Becker *et al.*, 1989) and random phosphorothioate oligodeoxynucleotides were synthesised on a multiple-column, automated DNA synthesiser (Applied Biosystems, Foster City, CA, USA) at 1.0 µmol synthesis scale. Oligomers were purified on denatured acrylamide gels, electroeluted and further purified by several cycles of precipitation with ethanol saturated with ammonium acetate; purity was also checked by reverse-phase HPLC. Concentrations of oligodeoxynucleotides were determined spectrophotometrically by absorbance at 260 nm, taking into account the molar extinction coefficient of the nucleotides present in each sequence. The random sequence oligodeoxynucleotide was used as a control in cell proliferation experiments.

*Bovine aortic endothelial cell cultures*

Cloned populations of endothelial cells were established from the intima of bovine aorta as described (Gospodarowicz *et al.*, 1976). Stock cultures were grown in 35 mm culture dishes in DMEM containing glucose ($1\ g\ l^{-1}$), H-FBS (10%), L-glutamine (2 mM), penicillin ($50\ IU\ ml^{-1}$) and streptomycin ($50\ \mu g\ ml^{-1}$); bFGF ($2\ ng\ ml^{-1}$) was added every other day. Cells were never allowed to reach confluency to avoid contact inhibition of growth and cells in exponential growth phase were either used for experiments or subcultured (up to ten subcultures) at a split ratio of 1:15. Cells were harvested by mild trypsinisation to minimise injury; monolayers were washed with PBS, dissociated with trypsin-EDTA in HBSS for 1–5 min at 4°C and finally washed with complete medium. Cells were resuspended in DMEM with supplements and cultured at 37°C, 5% $CO_2$ atmosphere. Cells were also evaluated for bFGF gene expression by Northern blot hybridisation. Total cellular RNA was extracted using the guanidine isothiocyanate-cesium chloride method (Sambrook *et al.*, 1989). RNA (30 μg/lane) was size-fractionated by 0.66 M formaldehyde-1.1% agarose gel elctrophoresis, transferred to nitrocellulose membranes, and hybridised by standard techniques to a $^{32}P$-labelled 25-mer oligodeoxynucleotide probe synthesised on the basis of a previous report (Abraham *et al.*, 1986). Each filter was also hybridised with a human γ-actin probe to normalise for the amounts of mRNA transferred. The blots were then autoradiographed for 16 h at − 75°C with intensifying screens. As positive control cell expressing the bFGF gene message, the DU145 prostate cancer cell line (ATCC, Rockville, MD, USA) was used (Nakamoto *et al.*, 1992).

Quiescent endothelial cells were obtained as described (Baldin *et al.*, 1990). Briefly, cells were seeded at low density ($10^3$ cells $cm^{-2}$) in 24-well plates in complete medium containing bFGF ($2\ ng\ ml^{-1}$); after 72 h they were washed twice with serum-free DMEM containing $Fe^{2+}$-saturated transferrin ($10\ \mu g\ ml^{-1}$) and cultures were continued in the same medium for 48 h. Cell cycle analysis was performed as reported (Pontzer *et al.*, 1991) to confirm the synchronisation of cells into $G_0G_1$ phase. Propidium iodide stained nuclei were analysed on a FACStar (Becton Dickinson, San Jose, CA, USA) using the CellFIT Cell-Cycle Analysis 2.0.2 software; in these conditions, 95% of cells were in $G_0G_1$ phase, 1.5% in S and 3.5% in $G_2M$ phase.

*Effects by bFGF, suramin and antisense bFGF on endothelial cell proliferation*

Cell proliferation was assayed by measuring the incorporation of [$^3$H]-thymidine into the DNA. $G_1 \rightarrow$ mitosis transition of quiescent endothelial cells was obtained by stimulation of cells with DMEM containing 0.4% H-FBS, 3.5 mg/100 ml BSA and bFGF (0.5, 1, 5, 10 and $50\ ng\ ml^{-1}$). Twenty-two hours after bFGF addition, cells were pulse-labelled for 2 h with $2\ \mu Ci\ ml^{-1}$ of [$^3$H]-thymidine. To terminate the reaction, cells were washed twice with ice-cold PBS, extracted with 10% (wt/vol) cold TCA and lysed with 0.25 N NaOH containing 4 mg/100 ml of salmon sperm DNA. Radioactivity was measured by resuspending 0.5 ml of the cell lysate in 10 ml of Ecoscint A (National Diagnostics, Manville, NJ, USA) and counted with a Packard 2000CA Tri-Carb liquid scintillation counter (Packard Instrument Co., Downers Grove, IL, USA). Flow cytometric analysis of exponentially growing cells in complete medium and of cells in serum-free culture stimulated by bFGF demonstrated that 60 and 75% of cells were in $G_0G_1$ phase, respectively. To evaluate the effect of suramin on basal and bFGF-induced DNA synthesis, $300\ \mu g\ ml^{-1}$ of suramin were added 6 h before bFGF; then the experiment was continued as reported above. The effect of sense and antisense bFGF oligodeoxynucleotides on endothelial cell proliferation was evaluated on cells in exponential growth phase treated for 2 days with oligodeoxynucleotides (10, 20 and 40 μM) which were added immediately after seeding and 24 h thereafter. At the end of the second day of treatment, cells were pulse-labelled for 2 h with $2\ \mu Ci\ ml^{-1}$ of [$^3$H]-thymidine and radioactivity was extracted and counted as reported above.

Furthermore, to assess the reversibility of the inhibition of cell proliferation by bFGF antisense oligodeoxynucleotide, bFGF ($10\ ng\ ml^{-1}$) was added 2 h after each antisense addition and cell proliferation was evaluated as reported above.

*Assay of angiogenesis in the chick chorioallantoic membrane model*

The shell-less embryo culture used in the present study is an adaptation of the procedure previously decribed (Vu *et al.*, 1985). Briefly, fertilised white Leghorn chick eggs were incubated for 3 days and then washed in Betadine® and 70% ethanol, cracked and the shell was separated from the embryos under a laminar flow hood. The embryos were placed in a plastic wrap-tripod apparatus modified from that described elsewhere (Dunn *et al.*, 1981). This consisted of a Petri dish cover, a plastic wrap/plexiglass midportion, and a humidifying plastic base. A Handiwrap plastic wrap (Dow Chemical Company, Indianapolis, IN, USA) was suspended within the chamber and formed a cradle for the contents of the egg; the humidifying base contained 1% $CuSO_4 \bullet 5H_2O$ to retard fungal growth. Four ml of MEM containing L-glutamine (2 mM), penicillin ($50\ IU\ ml^{-1}$) and streptomycin ($50\ \mu g\ ml^{-1}$) were added to the egg contents that were delivered to the assembled sterile culture chambers. Embryos were incubated at 37.5°C at a humidity of 50% for 3 days, at which point treatments to assay angiogenesis inhibition were started.

*Preparation of agarose disks and treatment of CAM*

Drugs were included into low-melting point agarose for sustained release, as previously reported (Dobson *et al.*, 1990). Agarose was dissolved and sterilised by autoclaving; the solution was cooled and kept at 38°C in a water bath, thereby avoiding any abnormal heating for the test drug to be added. Suramin, heparin and hydrocortisone were added and agarose solutions were poured on the surface of a 35 mm sterile plastic dishes and placed at 4°C to solidify. Agar was cut into round disks (diameter 5 mm and volume 25 μl) and peeled away from the dish by a sterile spatula. The concentration of agarose was 2.5% and each disk contained either suramin (25, 50 or 100 μg) or heparin (60 or 120 μg)/ hydrocortisone (50 or 100 μg). Disks were placed on the CAM of chick embryos which were returned to the incubator for 2 additional days. At the end of treatment, agarose disks were gently removed and assayed for drug release while embryos were sacrificed for quantitation of CAM vascular network.

*Quantitation of chick chorioallantoic membrane vascularisation*

The method described herein is an adaptation to the CAM of the procedure described in detail elsewhere (Proia *et al.*, 1988). After two days of treatment, a 20% fat emulsion for intravenous injection (Lipofundin 20, Braun Melsungen AG, Germany) was injected into chorioallantois of chicken embryos, so that the red colour of blood vessels contrasted sharply with the white colour; CAMs were photographed using a Polaroid model MP-4 camera system equipped with 105 mm objective.

Quantitation of angiogenesis inhibition was performed by an image analysis system. Briefly, each print of the same magnification was converted into a television image, and the digitised data were analysed as discrete values of varying shades of gray with an image analyser. This procedure allowed the resolution of faint boundaries of vessels; nonvascular noise was removed either by automatic cleaning process or by interactive use of an editor which could refine outlines of the boundaries. Components of the image analysis system were: image analyser ASBA (Wild + Leitz AG, F.R.G.), CD 233 monitor (BARC Industries, Belgium) and Polaroid freeze-frame video recorder. One $cm^2$ of the image array contained 1509 pixels and 270–290 different gray levels could be distinguished for each pixel. Using the digitiser tablet, a line was drawn to delineate the total perimeter of both CAM and treated area showing reduction of vascular network. Modification of blood vessel density due to treatments was estimated by a gray-scale analysis: the mean gray level of each treated area was computed and expressed as percent value compared to the mean gray level of the untreated CAM.

*Assessment of drug release from agarose disks*

Agarose disks were removed from the CAM at the end of treatment, gently homogenised with few strokes of a Dounce homogeniser with tight-fitting glass pestle at 550 rpm, and the volume of homogenate was brought to 0.3 ml with distilled water. Suramin was measured by a reverse-phase, ion-paring HPLC method (Supko & Malspeis, 1990) with fluorimetric detection. Heparin was assayed in samples by a colorimetric method (Khan & Newman, 1990) and the residual amount of hydrocortisone in disks was measured by a specific radioimmunologic method (Schiebinger *et al.*, 1986).

*Statistical analysis*

Results are given in the text as mean values unless otherwise specified. Data reported in graphs are mean values ± s.e.m. of *n* experiments. The effects of suramin and heparin/hydrocortisone on vascular network development and the resulting hypovascular area of CAM were compared with two-tailed Student's *t*-test for unpaired data (Zar, 1984). A *P* value less than 0.05 was considered to be significant.

### Results

*Modulation of endothelial cell growth by suramin, bFGF and bFGF antisense oligomer*

Northern blot analysis of total cellular RNA demonstrated the presence of bFGF transcripts (3.7 and 7 kb) in endothelial cells used in the present study (Figure 1). In quiescent cells (1.5% of cells in S phase), [$^3$H]-thymidine incorporation was stimulated when cells were exposed to bFGF; the increase peaked at 10 ng $ml^{-1}$ ( + 390% compared with controls) with 5% cells in S phase and remained considerably elevated at 50 ng/ml (Figure 2). Therefore, the effect of suramin on the mitogenic activity of bFGF on endothelial cells was evaluated. Suramin 300 μg $ml^{-1}$ added to the culture medium 6 h before bFGF almost completely inhibited the growth factor-induced cell proliferation (Figure 2). The effect of blocking the expression of bFGF gene was evaluated by using the antisense oligomer targeted against bFGF mRNA which was added to cell cultures in exponential growth phase. A 76% inhibition of [$^3$H]-thymidine incorporation into DNA was obtained after 48 h treatment with 10 μM of bFGF antisense oligomer (Figure 3). To demonstrate that its antiproliferative effect was sequence-specific, cells were exposed to the random sequence oligomer; under this condition, no significant inhibition of cell growth could be demonstrated. In addition to this, the inhibitory effect of antisense treatment was partially reversed by treatment with bFGF 10 ng $ml^{-1}$ (Figure 3).

*Angiogenesis inhibition in the chick chorioallantoic membrane*

Chick embryo survival until disk implantation was approximately 67% with a 14% loss during removal from the shell and a subsequent loss of 19% due to either infection or

**Figure 1** Northern blot analysis of bFGF transcripts in endothelial cells **a**, and positive control DU145 prostate cancer cells **b**. Total RNA (30 μg/lane) was size-fractionated on a 0.66 M formaldehyde-1.1% agarose gel electrophoresis, transferred to nitrocellulose membranes, and hybridised by standard techniques to the $^{32}$P-labelled oligomer probe. Blots were also probed with a human γ-actin probe **c**, to normalise for the amounts of RNA loaded. bFGF transcripts (3.7 and 7 kb) are shown by arrows.

**Figure 2** Stimulation of [$^3$H]-thymidine incorporation into DNA of endothelial cells by bFGF and its inhibition by suramin. Quiescent cells were stimulated with D-MEM containing 0.4% H-FBS, 3.5 mg/100 ml BSA and bFGF 0.5, 1, 5, 10 and 50 ng $ml^{-1}$ (white circles). Twenty-two hours after bFGF addition, cells were pulse-labelled for 2 h with 2 μCi $ml^{-1}$ of [$^3$H]-thymidine. Radioactivity was measured in TCA precipitable material by liquid scintillation counting. In experiments involving suramin (black circles), the drug (300 μg $ml^{-1}$) was added to the culture medium 6 h prior to bFGF.

abnormal differentiation, in agreement with an earlier published report (Woltering *et al.*, 1991). After 2 days of treatment, the branching pattern of blood vessels below disks containing heparin 60 μg/hydrocortisone 50 μg was reduced (Figure 4a); based on the gray-scale analysis, the mean

**Figure 3** Inhibition of [$^3$H]-thymidine incorporation into DNA of endothelial cells by antisense oligomer against bFGF mRNA and reversal by bFGF. Cells in exponential growth phase were treated twice at 24 h intervals with graded concentrations of the antisense (white squares) and random (white circles) oligomers. At the end of the second day of treatment, cells were pulse-labelled for 2 h with 2 $\mu$Ci ml$^{-1}$ of [$^3$H]-thymidine and radioactivity was measured into TCA precipitable material by liquid scintillation counting. bFGF 10 ng ml$^{-1}$ (black squares) was added 2 h after each oligomer addition and cell proliferation was evaluated as reported above.

absolute values of vascular densities of control and treated CAMs were 112 and 79 respectively, which corresponded to a 29% decrease over an area of 0.6 cm$^2$ (Figure 5). In the CAM around the disks containing suramin 50 $\mu$g the tiny vessel loops were absent (Figure 4b) and the mean gray-scale value was 72, corresponding to a 35% inhibition over a surface of 2.4 cm$^2$ (Figure 5). In both treatments, the faint boundaries and capillaries of CAM were severely affected, while the larger vessels were reduced in caliber. The reduction in vascular density was slightly more pronounced in suramin-treated CAMs, but the difference with heparin/hydrocortisone was not significant (Figure 5). However, the area showing a decrease in vascularisation was significantly larger after exposure to suramin than to heparin/hydrocortisone (Figure 5). A lower amount of suramin (25 $\mu$g/disk) produced a modest antiangiogenic effect; the mean gray-scale value was 95 indicating a 15% reduction of vascular density compared to controls and the mean value of the area with reduced vascular density was 0.8 cm$^2$. Higher amounts of suramin (100 $\mu$g) or heparin (120 $\mu$g)/hydrocortisone (100 $\mu$g) included in agarose disks were associated with a marked reduction in chick embryo survival (less than 2 days) due to thrombosis, hemorrhage or marked distortion of large vessels. Therefore, in our analysis, suramin 50 $\mu$g or heparin 60 $\mu$g/hydrocortisone 50 $\mu$g represented the optimum doses with maximum antiangiogenic activity and embryo survival. Moreover, microscopic examination of CAMs treated with suramin 50 $\mu$g did not reveal thrombi in large vessels. The reason for the decrease in vascularisation and the disappearance of capillaries (thrombosis or failure to form blood vessels) could not be demonstrated. In each case, if the agarose disks were removed and the CAMs were allowed to recover for 2–4 days, the avascular area was substantially unchanged with respect to vascular density and surface (data not shown). The area of CAM below control disks without drugs did not show changes in vascular density with a normal leaf-life branching pattern of blood vessels (Figure 4c), indicating that the disk weight did not affect their growth during the experiment.

**Figure 4** Computer-generated images derived from CAMs after a 2-day treatment with agarose disks containing heparin 60 $\mu$g/hydrocortisone 50 $\mu$g **a**, suramin 50 $\mu$g **b**, or vehicle control **c**. The white colour corresponds to the CAM with reduction of blood vessels density (circumferential arrowheads) while the gray colour corresponds to the normal CAM. In each case, the position and dimension of the agarose disks are identified by small rings. The bar at the bottom of each picture corresponds to 1 cm.

Agarose disks provided an inert support for drug delivery; at the end of their permanence on the CAM, the mean percentage value of drug released was 54.6, 80.2, and 60.4 for heparin, hydrocortisone and suramin, respectively (Table I).

**Figure 5** Decrease in vascular density and extension of the area of CAM treated with suramin 50 μg (white bars) or heparin 60 μg/hydrocortisone 50 μg (black bars). Differences in vascular density of treated *vs* untreated CAMs were calculated by analysing the digitised data as discrete values of varying shades of gray with the aid of an image analysis system; the same system allowed the measurement of the area with reduced blood vessel density. $^*P<0.05$.

### Discussion

The antitrypanosomal drug suramin selectively dissociates several heparin-binding growth factors from their receptors and produces antiproliferative effects on cancer cells both *in vitro* and *in vivo* (for review see La Rocca *et al.*, 1990). These effects were proposed to occur by inhibition of growth factor receptor binding or by a modified interaction between growth factor receptor and autosecreted oncogene products (Moscatelli & Quarto, 1989). bFGF is an heparin-binding mitogen that plays a central role in tumour development and metastatis (Becker *et al.*, 1989). It affects the growth of many cell types *via* high affinity membrane receptors and is a potent endothelial growth factor capable of inducing the formation of new capillary blood vessels *in vivo* at nanogram amounts (Risau *et al.*, 1990). Inhibition of angiogenesis by means of bFGF immunoneutralising monoclonal antibody has been reported to be associated with antitumour effect *in vivo* (Hori *et al.*, 1991), suggesting that novel therapeutic approaches to cancer therapy might involve the use of antiangiogenic drugs.

The results of the present study indicate that bFGF gene message is detectable in the endothelial cells and that cells have bFGF as a mitogenic factor. Further evidence on the possible autocrine role of bFGF on endothelial cells is suggested in the present study where an antisense oligomer targeted against bFGF mRNA, a specific tool of inhibition of gene expression (Helene & Toulmé, 1990), markedly reduces the [$^3$H]-thymidine incorporation in exponentially growing cells. The effect was partially inhibited by the subsequent addition of exogenous bFGF in the culture medium. Suramin and antisense oligomers appear to be drugs acting at two different levels of bFGF mitogenic activity; suramin acts primarily at the level of cell surface receptors, while the antisense oligomer exerts its effect intracellularly at the level of mRNA processing (Helene & Toulmé, 1990).

The biologic activity of bFGF is the result of the interaction of the growth factor released in the endoplasmic reticulum with specific receptors or the secretion of bFGF in the extracytoplasmic compartment and binding to cell surface receptors. Even if bFGF lacks the secretory signal sequence and is primarily retained intracellulary, bFGF is also released in the extracellular compartment (Mignatti & Rifkin, 1991). Suramin at a concentration of 300 μg ml$^{-1}$, a clinically effective drug level, is able to suppress bFGF proliferative activity on endothelial cells. At least two previously published works describe the inhibitory effect of suramin on bFGF biologic activity (Sato & Rifkin, 1988; Moscatelli & Quarto, 1989) and one of them describes the effects of bFGF on bovine endothelial cell motility and DNA synthesis. The data of the above-mentioned reports, however, were obtained with: (i) a very high level of suramin (0.75–1 mM corresponding to 1.1–1.4 mg ml$^{-1}$), and (ii) a low protein concentration (1% FBS) in the culture medium. Suramin plasma levels in patients should not exceed 300 μg ml$^{-1}$ (approximately 0.21 mM) (La Rocca *et al.*, 1990) to reduce the risk of severe neurotoxicity and many of the drug's biological properties (e.g. the antiproliferative activity) are dependent on protein concentration in culture medium. This is due to its high affinity for plasma proteins: more than 90% of the drug is bound to albumin and other globulins (Hawking, 1978). As we gain knowledge in the clinical use of suramin as a chemotherapeutic agent, there is a need for a re-evaluation of suramin's pharmacodynamic properties under adequate experimental conditions. In the present study, the results were obtained in culture medium supplemented with 10% FBS and with up to 5-fold less suramin concentration than that used in previous studies.

The results presented herein demonstrate that after a 2-day exposure to a single dose of suramin 50 μg/disk, the density of normal developing vascular network in the CAM of chick embryos was decreased by 35%; this effect was also produced by heparin 60 μg/hydrocortisone 50 μg ( – 29% compared to normal CAM) and the difference between treatments was not significant. Whether this effect is the result of a specific inhibition of bFGF remains to be demonstrated. The evidence that both suramin and heparin/hydrocortisone inhibit the growth of blood vessels suggests that heparin-binding angiogenic factors play an important role in stimulating the growth of blood vessels in the CAM. The major difference between treatments consisted of the extension of the hypovascular area; that under disks containing suramin was significantly larger than that under heparin/hydrocortisone. This effect might be dependent on the diffusion of suramin out of the agarose disks and on the resistance of the drug to metabolic degradation by living organisms (La Rocca *et al.*, 1990). On the contrary, the effect of heparin/hydrocortisone seems to be confined to the close proximity of the disks; this effect might be dependent on degradation by cells or dilution of the drugs under concentrations critical to their activity. The amount of drugs delivered by disks showed that suramin was even more effective than heparin/hydrocortisone, since the released amount of the former drug was 29.1 ± 2.2 μg while that of the latter combination was 34.1 ± 4.8 and 41.6 ± 3.6 μg for heparin and hydrocortisone, respectively. Due to the absence of inflammatory reactions around the agarose disks and the normal development of blood vessels under them, the present study provide evidence that this support is a useful tool for

**Table I** Release of heparin, hydrocortisone and suramin from agarose disks used for delivering drugs to the CAM[a]

| *Drug (μg)* | *Before treatment* | *After treatment* | *Drug released* | *Mean release (%)* |
|---|---|---|---|---|
| Heparin | 62.5 ± 4.6 | 28.4 ± 3.9 | 34.1 ± 4.8 | 54.6 |
| Hydrocortisone | 51.9 ± 2.9 | 10.3 ± 1.2 | 41.6 ± 3.6 | 80.2 |
| Suramin | 48.2 ± 5.3 | 19.1 ± 1.1 | 29.1 ± 2.2 | 60.4 |

[a]Disks were applied on the CAM of chick embryos and removed 2 days later; drug content (μg) was assayed in $n = 10$ disks for each treatment as reported in the Materials and methods section delivering drugs to the CAM. The angiostatic effect of heparin, steroids and suramin was recently demonstrated in a model of glass-fiber filter stimulated angiogenesis in the CAM (Wilks *et al.*, 1991). Suramin and heparin alone had a modest angiostatic effect on the CAM; however the most active single compound was cortisone acetate, followed by other steroids. The addition of heparin or suramin to the steroids produced an increase in the angiogenesis inhibition (Wilks *et al.*, 1991). Drug release from the inert support used for drug administration, however, was not measured, thus the comparison of the angiostatic effects might suffer from the lack of these data. In the present study no angiogenesis inhibition was found using heparin alone; only the addition of hydrocortisone elicited the angiostatic effect of heparin, in agreement with earlier reports (Folkman *et al.*, 1983; Maragoudakis *et al.*, 1989; Lee *et al.*, 1990). This might be dependent on the different experimental conditions; the angiogenesis induced in the CAM by glass-fiber filters (Wilks *et al.*, 1991) was inhibited by cortisone acetate, an agent used by other authors to prevent the phlogistic reaction in the CAM following the experimental manipulation (Maragoudakis *et al.*, 1989) and might be dependent at least in part on the release of mediators of inflammation.

In conclusion, the present report shows that suramin is an effective inhibitor of bFGF mitogenic activity in endothelial cells and of angiogenesis in the CAM, suggesting an additional mechanism of the pharmacological activity of the drug which could be relevant to its antitumour activity.

This work was supported in part by a generous contribution from the Italian Association for Cancer Research (AIRC, Milano, Italy).

### References

ABRAHAM, J.A., MERGIA, A., WHANG, J.L., TUMOLO, A., FRIEDMAN, J., HJERRILD, K.A., GOSPODAROWICZ, D. & FIDDES, J.C. (1986). Nucleotide sequence of a bovine clone encoding the angiogenic protein, basic fibroblast growth factor. *Science*, **233,** 545–548.

BALDIN, V., ROMAN, A.-M., BOSC-BIERNE, I., AMALRIC, F. & BOUCHE, G. (1990). Translocation of bFGF to the nucleus is G1 phase cell cycle specific in bovine aortic endothelial cells. *EMBO J.*, **9,** 1511–1517.

BECKER, D., MEIER, C.B. & HERLYN, M. (1989). Proliferation of human malignant melanomas is inhibited by antisense oligodeoxynucleotides targeted against basic fibroblast growth factor. *EMBO J.*, **8,** 3685–3691.

COFFEY, R.J. Jr, LEOF, E.B., SHIPLEY, G.D. & MOSES, H.L. (1987). Suramin inhibition of growth factor receptor binding and mitogenicity in AKR-2B cells. *J. Cell. Physiol.*, **132,** 143–148.

DOBSON, D.E., KAMBE, A., BLOCK, E., DION, T., LU, H., CASTELLOT, J.J. Jr. & SPIEGELMAN, B.M. (1990). 1-Butyryl-glycerol: a novel angiogenesis factor secreted by differentiating adipocytes. *Cell*, **61,** 223–230.

DUNN, B.E., FITZHARRIS, T.P. & BARNETT, B.D. (1981). Effects of varying chamber construction and embryo pre-incubation age on survival and growth of chick embryos in shell-less culture. *Anat. Rec.*, **199,** 33–43.

FOLKMAN, J., LANGER, R., LINHARDT, R.J., HAUDENSCHILD, C. & TAYLOR, S. (1983). Angiogenesis inhibition and tumor regression caused by heparin or a heparin fragment in the presence of cortisone. *Science*, **111,** 719–725.

FOLKMAN, J. (1990). What is the evidence that tumours are angiogenesis-dependent? *J. Natl Cancer Inst.*, **82,** 4–6.

GAGLIARDI, A. & COLLINS, D.C. (1993). Inhibition of angiogenesis by antiestrogens. *Cancer Res.*, **53,** 533–535.

GOSPODAROWICZ, D., MORAN, J., BRAUN, D. & BIRDWELL, C. (1976). Clonal growth of bovine vascular endothelial cells: fibroblast growth factor as a survival agent. *Proc. Natl Acad. Sci. USA*, **73,** 4120–4124.

HAWKING, F. (1978). Suramin: with special reference to onchocerciasis. *Adv. Pharmacol. Chemother.*, **15,** 289–322.

HAYEK, A., CULLER, F.L., BEATTIE, G.M., LOPEZ, A.D., CUEVAS, P. & BAIRD, A. (1987). An *in vivo* model for study of the angiogenic effects of basic fibroblast growth factor. *Biochem. Biophys. Res. Commun.*, **147,** 876–880.

HELÈNE, C. & TOULME, J.-J. (1990). Specific regulation of gene expression by antisense, sense and antigene nucleic acids. *Biochim. Biophys. Acta*, **1049,** 99–125.

HORI, A., SASADA, R., MATSUTANI, E., NAITO, K., SAKURA, Y., FUJITA, T. & KAZAI, Y. (1991). Suppression of solid tumor growth by immunoneutralizing monoclonal antibody against human basic fibroblast growth factor. *Cancer Res.*, **51,** 6180–6184.

INGBER, D., FUJITA, T., KISHIMOTO, S., SUDO, K., KANAMARU, T., BREM, H. & FOLKMAN, J. (1990). Synthetic analogues of fumagillin that inhibit angiogenesis and suppress tumor growth. *Nature*, **348,** 555–557.

KHAN, M.Y. & NEWMAN, S.A. (1990). An assay for heparin by decrease in color yield (DECOY) of a protein-dye-binding reaction. *Anal. Biochem.*, **187,** 124–128.

LA ROCCA, R.V., STEIN, C.A., DANESI, R., MYERS, C.E. (1990). Suramin, a novel antitumour compound. *J. Steroid Biochem. Mol. Biol.*, **37,** 893–898.

LEE, J.K., CHOI, B., SOBEL, R.A., CHIOCCA, E.A. & MARTUZA, R.L. (1990). Inhibition of growth and angiogenesis of human neurofibrosarcoma by heparin and hydrocortisone. *J. Neurosurg.*, **73,** 429–435.

MAIONE, T.E. & SHARPE, R.J. (1990). Development of angiogenesis inhibitors for clinical applications. *Trends Pharmacol. Sci.*, **11,** 457–461.

MARAGOUDAKIS, M.E., SARMONIKA, M. & PANOUTSACOPOULOU, M. (1989). Angiogenic action of heparin plus cortisone is associated with decreased collagenous protein synthesis in the chick chorioallantoic membrane system. *J. Pharmacol. Exp. Ther.*, **251,** 679–682.

MIGNATTI, P. & RIFKIN, D.B. (1991). Release of basic fibroblast growth factor, an angiogenic factor devoided of secretory signal sequence – a trivial phenomenon or a novel secretion mechanism. *J. Cell. Biochem.*, **47,** 201–207.

MOSCATELLI, D. & QUARTO, N. (1989). Transformation of NIH 3T3 cells with basic fibroblast growth factor or the hst/K-*fgf* oncogene causes downregulation of the fibroblast growth factor receptor: reversal of morphological transformation and restoration of the receptor number by suramin. *J. Cell. Biol.*, **109,** 2519–2527.

MYERS, C., COOPER, M., STEIN, C., LA ROCCA, R., MCCLELLAN, M.W., WEISS, G., CHOYKE, P., DAWSON, N., STEINBERG, S., UHRICH, M.M., CASSIDY, J., KOHLER, D.R., TREPEL, J. & LINEHAN, M.W. (1992). Suramin: a novel growth factor antagonist with activity in hormone-refractory metastatic prostate cancer. *J. Clin. Oncol.*, **10,** 881–889.

NAKAMOTO, T., CHANG, C., LI, A. & CHODAK, G.W. (1992). Basic fibroblast growth factor in human prostate cancer cells. *Cancer Res.*, **52,** 571–577.

PONTZER, C.H., BAZER, F.W. & JOHNSON, H.M. (1991). Antiproliferative activity of a pregnancy recognition hormone, ovine trophoblast protein-1. *Cancer Res.*, **51,** 5304–5307.

PROIA, A.D. CHANDLER, D.B., HAYNES, W.L., SMITH, C.F., SUVARNAMANI, C., ERKEL, F.H. & KLINTWORTH, G.K. (1988). Quantitation of corneal neovascularization using computerized image analysis. *Lab. Invest.*, **58,** 473–479.

RISAU, W. (1990). Angiogenic growth factors. *Progr. Growth Factor Res.*, **2,** 71–79.

SAMBROOK, J., FRITSCH, E.F. & MANIATIS, T. (1989). *Molcular Coning – A Laboratory Manual.* 2nd Edn. Cold Spring Harbor Laboratory Press: Cold Spring Harbor.

SATO, Y. & RIFKIN, D.B. (1988). Autocrine effect of basic fibroblast growth factor: regulation of endothelial cell movement, plasminogen activator synthesis, and DNA synthesis. *J. Cell. Biol.*, **107,** 1199–1205.

SCHIEBINGER, R.J., CHROUSOS, G.P., CUTLER, G.B. Jr & LORIAUX D.L. (1986). The effect of serum prolactin on plasma adrenal androgens and the production and metabolic clearance rate of dehydroepiandrosterone sulfate in normal and hyperprolactinemic subjects. *J. Clin. Endrocrinol. Metab.*, **62,** 202–209.

SCHREIBER, A.B., WRINKLER, M.E., DERYNCK, R. (1986). Transforming growth factor-α; a more potent angiogenic mediator than epidermal growth factor. *Science*, **233,** 1250–1253.

STEIN, C.A., LA ROCCA, R.V., THOMAS, R., MCATEE, N. & MYERS, C.E. (1989). Suramin: an anticancer drug with a unique mechanism of action. *J. Clin. Oncol.*, **7,** 499–508.

SUPKO, J.G. & MALSPEIS, L. (1990). A rapid isocratic HPLC assay of suramin (NSC 34936) in human plasma. *J. Liquid Chromatogr.*, **13**, 727–741.

VU, M.T., SMITH, C.F., BURGER, P.C. & KLINTWORTH, G.K. (1985). An evaluation of methods to quantitate the chick chorioallantoic membrane assay in angiogenesis. *Lab. Invest.*, **53**, 499–508.

WEIDNER, N., SEMPLE, J.P., WELCH, W.R. & FOLKMAN, J. (1991). Tumor angiogenesis and metastasis. Correlation in invasive breast carcinoma. *N. Engl. J. Med.*, **324**, 1–8.

WILKS, J.W., SCOTT, P.S., VRBA, L.K. & COCUZZA, J.M. (1991). Inhibition of angiogenesis with combination treatments of angiostatic steroids and suramin. *Int. J. Radiat. Biol.*, **60**, 73–77.

WISEMAN, D.M., POLVERINI, P.J., KAMP, D.W. & LEIBOWICH, S.J. (1988). Transforming growth factor $\beta$ (TGF-$\beta$) is chemotactic for human monocytes and induces their expression of angiogenic activity. *Biochem. Biophys. Res. Commun.*, **157**, 793–800.

WOLTERING, E.A., BARRIE, R., O'DORISIO, T.M., ARCE, D., URE, T., CRAMER, A., HOLMES, D., ROBERTSON, J. & FASSLER, J. (1991). Somatostatin analogues inhibit angiogenesis in the chick chorioallantoic membrane. *J. Surg. Res.*, **50**, 245–251.

YAYON, A. & KLAGSBRUN, M. (1990). Autocrine transformation by chimeric signal peptide-basic fibroblast growth factor: reversal by suramin. *Proc. Natl Acad. Sci. USA*, **87**, 5346–5350.

ZAR, J.H. (1984). *Biostatistical Analysis*. Prentice-Hall: Englewood Cliffs.